# Peptide-Based Vaccines for Neurodegenerative Diseases: Recent Endeavors and Future Perspectives

**DOI:** 10.3390/vaccines9111278

**Published:** 2021-11-04

**Authors:** Vyronia Vassilakopoulou, Chrysoula-Evangelia Karachaliou, Alexandra Evangelou, Christos Zikos, Evangelia Livaniou

**Affiliations:** 1 Immunopeptide Chemistry Lab., Institute of Nuclear & Radiological Sciences & Technology, Energy & Safety (INRASTES), National Centre for Scientific Research “Demokritos”, Patr. Grigoriou E’ & 27 Neapoleos St., 15341 Agia Paraskevi, Greece; xrisak15@rrp.demokritos.gr (C.-E.K.); chzikos@yahoo.gr (C.Z.); 2 Department of Wine, Vine and Beverage Sciences, University of West Attica, 12243 Athens, Greece; aevangelou@uniwa.gr

**Keywords:** vaccines, neurodegenerative diseases, Alzheimer’s disease, beta-amyloid peptide, tau protein, α-synuclein, peptide epitopes, vaccine formulation, clinical trials, animal preclinical studies

## Abstract

The development of peptide-based vaccines for treating human neurodegenerative diseases has been the eventual aim of many research endeavors, although no active immunotherapies have been approved for clinical use till now. A typical example of such endeavors is the effort to develop vaccines for Alzheimer’s disease based on the beta-amyloid peptide, which continues to be intensively investigated despite previous setbacks. In this paper, recent developments in peptide-based vaccines which target beta-amyloid as well as tau protein and α-synuclein are presented. Particular focus has been directed toward peptide epitopes and formulation systems selected/developed and employed to enhance vaccine efficacy and safety. Results from both, human clinical trials and animal preclinical studies conducted mainly in transgenic mice have been included. Future perspectives on the topic are also briefly discussed.

## 1. Introduction

Neurodegenerative diseases are associated with a progressive loss of neurons in the central nervous system (CNS) and are characterized by severe clinical deficits, especially cognitive, motor, and psychiatric ones. Since aging is among the strongest risk factors causing their development, neurodegenerative diseases are considered a big public health problem with high socioeconomic impact in the developed world, where people’s life span has been continuously increased during the last decades. The most common neurodegenerative disease is Alzheimer’s disease (AD), while other well-known neurodegenerative diseases include Parkinson’s disease (PD), amyotrophic lateral sclerosis (ALS), Huntington’s disease (HD), etc. [1].

AD, first described by Dr. Alois Alzheimer in 1906 [2], is clinically characterized by memory, psychological and psychiatric deficits as well as language impairment, which progressively lead to practical difficulties in executing a normal everyday life and, eventually, to death [3,4]. As estimated, people with AD worldwide are currently around 40 million and this number is expected to continuously grow. AD and neurodegenerative diseases, in general, are complex and very heterogeneous. Aging seems to be the main risk factor, since the occurrence of AD increases substantially with age and becomes almost double every 5 years after the age of 65 [5]. Nevertheless, other reasons that may cause AD have been reported in the literature, such as abnormal nutrition, malfunction of the immune system, infectious agents, serious brain damage and degeneration of crucial anatomical pathways, mitochondrial dysfunction that may cause metabolic disorders, vascular factors that may affect the functioning of the blood–brain barrier, or exposure to environmental toxic substances [6,7]. On the other hand, specific genetic abnormalities, including mutations of amyloid precursor protein and presenilin genes as well as allelic variation in apolipoprotein E (ApoE), have also been associated with AD.

AD appears in two forms: sporadic AD, which is late-onset, i.e., symptoms begin after the age of 65, and represents the vast majority of AD cases (~95%), and familial AD, an early-onset and dominantly inherited disease, which accounts for a low percentage (~5%) of AD cases [5]. Although age is the major risk factor for sporadic AD, the presence of ApoE-ε4 genetic polymorphism highly increases the possibilities of developing the disease in persons older than 65 years [8]. Moreover, a large genome meta-analysis of clinically diagnosed late-onset AD cases confirmed 20 previously reported risk loci and also identified some new ones [5], associated e.g., with lipid metabolism and immunity [3]. On the other hand, familial AD affects younger individuals and is associated with mutated forms of the amyloid precursor protein (APP) and the core proteins of γ-secretase complex, presenilin 1 and presenilin 2 (PS1 and PS2), the presence/function of which result in accumulation of amyloid β peptide (Aβ) and formation of extracellular amyloid plaques [8,9]. In general, according to the well-known “amyloid cascade hypothesis” [10], under certain circumstances neurotoxic Aβ can be produced, accumulated/aggregated, and ultimately damage neurons, thus leading to dementia [3]. Though the pathogenesis of AD has not yet been completely understood, the amyloid cascade hypothesis remains the best known and most widely accepted theory proposed until now. In addition to the extracellular amyloid plaques composed of Aβ, AD is also characterized by the formation of intracellular neurofibrillary tangles composed of the microtubule-associated protein tau in an abnormally phosphorylated form [11]. Thus, abnormal Aβ and tau aggregates have been considered the most typical characteristic of AD [1,12]. Though it is not clear how these aberrant oligomers/aggregates can lead to neurodegeneration, synaptic dysfunction and neuroinflammation they seem to cause might be the reason [13,14]. One other protein, i.e., α-synuclein (α-syn), has been initially associated with another well-known neurodegenerative disease, i.e., PD, along with specific neurodegenerative diseases, such as dementia with Lewy bodies (DLB); α-syn is the main component of Lewy bodies, i.e., the most evident histopathological characteristic of PD and all of the aforementioned diseases [15,16]. In addition to PD, α-syn has been lately associated with AD, too [17]. Misfolding/accumulation of additional proteins, e.g., superoxide dismutase 1 and huntingtin, in specific brain regions has been related to the onset/progress of neurodegenerative diseases, such as ALS and HD, respectively [1,14,18]. To further support the linkage between certain proteins/peptides and neurodegenerative diseases, focusing on AD, we should mention that cerebrospinal fluid (CSF) levels of Aβ- and tau- related biomarkers have been widely employed for confirming AD diagnosis, along with positive amyloid-PET scans [5,19]. Moreover, pathological features related to Lewy bodies, mainly composed of α-syn, have been reported to occur in brains of patients with AD, while the elevation of α-syn CSF- levels in AD patients has been associated with clinical decline [17]. 

Treatment of AD is a very important issue for health care systems [5]. Until recently, treatment for AD patients relied on the pharmacological intervention of cholinergic and glutamatergic neurotransmission. Thus, a few drugs for AD have been approved by the US Food and Drug Administration (FDA), including donepezil, rivastigmine, and galantamine (cholinesterase inhibitors), memantine (an N-methyl-D-aspartate receptor partial antagonist), and a combination of donepezil and memantine. All these drugs, however, have limited effectiveness and they do not reverse the progression of the disease or improve cognitive dysfunction, but they rather delay the deterioration of AD symptoms for a limited time period [8]. Recently, a fully human monoclonal antibody targeting Aβ, aducanumab, has been approved by the FDA, although there has been some controversy concerning the overall efficacy of this passive immunotherapeutic agent [20,21,22,23,24]. Thus, it is of utmost importance to investigate and develop further therapeutic/preventive strategies to fight AD and other neurodegenerative diseases [3]. 

Vaccination is a highly effective strategy followed in the Public Health Sector as a means to defeat especially infectious diseases. Vaccines induce immunogen-specific clones of B and T lymphocytes, which recognize different regions of the immunogen (usually a protein or a suitable protein-conjugate), i.e., peptide epitopes. B cells recognize “exposed” parts of the immunogen, which may be linear or conformational and are known as B cell epitopes, while T cells recognize linear peptide sequences of the immunogen (T cell epitopes). Differentiation of B cells into mature plasma cells leads to the production of large amounts of antibodies, which recognize the same B-cell epitope that has caused B-cell stimulation in the first place; B and T cells may differentiate into memory cells, which ensure enhanced immune response after subsequent exposure to the same immunogen. Several attempts to exploit the power of the immune system and develop active immunotherapies (i.e., vaccines) against AD have been made during the last twenty years [5], while passive immunotherapy strategies, in which specific antibodies against the biomolecular target, such as aducanumab, are directly administered to the host organism have also been followed, in the frame of preclinical studies and/or clinical trials with human subjects [14]. Immunization against Aβ as an active immunotherapy for AD has its origins in the idea that antibodies targeting Aβ may block its aggregation and, consequently, prevent the disease progression. One should take into consideration, however, that Aβ is a self-peptide that may either be non-immunogenic or induce a damaging autoimmune reaction [5] and, thus, efficacy and safety of active immunotherapies for AD are major issues to be addressed. Nevertheless, and despite no vaccine for AD has been approved till now, the basic concept is still considered promising and several vaccine candidates with improved characteristics are being currently studied [5]. All Aβ-vaccines so far reported may be considered peptide-based vaccines, since Aβ consists of less than 45 amino acids (see Section 2.2.1) and is, therefore, thought to be a peptide rather than a protein [25]; on the other hand, vaccines targeting tau and α-syn can be considered peptide-based, if specific peptide epitopes, instead of the intact parental protein, have been employed for their development.

The goals of the present work can be summarized as follows: (i) to underline the continuous interest in the field of developing active immunotherapy approaches based on appropriately selected/designed peptide epitopes of specific biomolecular targets (Aβ, tau and α-syn) which have been associated with neurodegenerative diseases, (ii) to list the most important endeavors in this field, including recent studies, not mentioned in previous review papers, (iii) to highlight specific factors, e.g., the formulation systems used or the experimental conditions employed during preclinical evaluation (animal models/vaccination schemes, etc.), which may affect the final outcome. Future perspectives in the field are also briefly mentioned.

## 2. Aβ, Tau, and α-Syn as Biomolecular Targets of Peptide-Based Vaccines for Neurodegenerative Diseases

### 2.1. Biomolecular Targets of Vaccines for Neurodegenerative Diseases

Significant efforts toward the development of active immunotherapies/vaccines for neurodegenerative diseases have focused on targeting pathologic species of Aβ, tau, and, to a lesser extent α-syn since aberrant aggregation of these biomolecules is considered to play a major role in the disease pathophysiology [26]. Immunization targeting these peptides/proteins has been expected to result in the generation of antibodies that might facilitate clearance or prevent the formation of neurotoxic forms of the parental targets [4]. However, there are some issues that should be taken into account and appropriately addressed, in order to achieve optimal results: As already mentioned, Aβ, as well as tau and α-syn, are probably conceived as “self” molecules by the host organism; thus, they may be either non-immunogenic or induce a detrimental auto-immune reaction [5] and this aspect should be carefully taken into consideration when designing such a vaccine. Another issue is how the antibodies induced after immunization can cross the blood–brain barrier and access the pathologic spots [4]. However, antibodies against Aβ that had been peripherally administered were reported to enter CNS in mouse models of AD, and in human subjects participating in clinical trials [5,27]. In vivo access of the pathologic loci is even more complicated, when intracellular proteins are targeted, such as tau. Previous experimental data have shown, however, that intracellular “neurodegenerative” proteins may be also present extracellularly [14]. Moreover, recent technological advances, especially in the field of delivery systems, have facilitated successful in vivo accessibility of pathologic targets and stimulated new efforts to develop active immunotherapies targeting Aβ, tau, and α-syn [14].

According to recently accumulated evidence, neurotoxic species of Aβ, tau, and α-syn may exert their toxicity at least in part through binding to cellular prion protein (PrP) on the surface of neurons [28,29,30]. Thus, PrP rather than individual disease-associated proteins might be (immuno)therapeutically targeted. Moreover, a few additional molecular targets have been reported in the literature, e.g., TAR DNA-binding protein 43 (TDP-43), dipeptide repeat proteins (DPRs), superoxide dismutase 1, and huntingtin protein [1,14,18,31,32], but little data on the development of vaccines against these targets are available.

### 2.2. Aβ, Tau, and α-Syn as Targets of Vaccines for Neurodegenerative Diseases

#### 2.2.1. Aβ

Aβ, which is the main component of the extracellular amyloid plaques in the AD brain, exists in two main forms, composed of 40 amino acids [Aβ(1-40)] and 42 amino acids [Aβ(1-42)], respectively [3]. Aβ is formed through proteolysis of APP by β- and γ-secretase. More specifically, APP, which is a membrane protein occurring in many tissues and particularly concentrated in neuronal synapses, undergoes proteolytic degradation by α-, β-, and γ-secretases. APP proteolysis by α- secretase leads to products that are not amyloidogenic or toxic, while proteolysis by β- and subsequently γ- secretase results in the formation of the amyloidogenic APP fragments, Aβ(1-40) and Aβ(1-42), the latter of which seems to aggregate more readily and be more toxic [3]. Mutations in APP or APP gene duplication as well as mutations in the core proteins of γ-secretase complex, PS1 and PS2, may increase Aβ aggregation into oligomers, amyloid fibrils, and plaques, all of which can be cytotoxic [3]. In general, according to the amyloid cascade hypothesis, an imbalance between formation and clearance of Aβ is considered critical for developing AD [4,5,10]. The exact mechanisms through which different Aβ species contribute to neurotoxicity remain largely unknown, but recent evidence suggests that soluble oligomers are the most neurotoxic [4]. Structures/shapes of Aβ monomers, oligomers, and fibrils have been extensively studied in the last few years with various experimental techniques [33].

#### 2.2.2. Tau

Although pathologic Aβ is the prevalent toxic event in AD according to the amyloid cascade hypothesis, clearance of Aβ alone may be insufficient to stop/delay the disease progress especially in the later stages of AD, while accumulating evidence shows that pathologic tau may also correlate with disease pathogenesis and progression [34]. Interestingly, there may be a link between Aβ and tau in AD pathophysiology, since Aβ(1-42) oligomers have been reported to cause, among other toxic effects, tau hyperphosphorylation [5]. Tau is a microtubule-associated protein that is highly expressed in neurons. There are six isoforms of tau protein, which vary in size from 352 to 441 amino acid residues. The different isoforms contain three or four tubulin-binding repeats (3R, 4R) also known as microtubule-binding repeats (MTBRs) of 31 or 32 amino acids each in their C-terminus and one, two, or no inserts of 29 amino acids each in the N-terminus of the molecule. Primary sequence analysis has shown that tau consists of four distinct regions: the N-terminal domain (NTD), the proline-rich domain (PRD), the microtubule-binding region (MTBR) and the C-terminal domain (CTD). Two aggregation-prone hexapeptide motifs have been identified, (275-280) and (306-311), which are located on the R2 repeat and R3 repeat of MTBR, respectively [35]. Tau filaments from AD have been studied with cryogenic electron microscopy [36]; moreover, filaments formed in vitro from the truncated tau(297-391) have been reported to resemble paired helical filaments found in AD brains at the macromolecular level [37]. Tau is post-translationally modified through phosphorylation and bears around 80 potential phosphorylation sites, which are targets of several kinases and phosphatases [4]. Hyperphosphorylation, as well as truncation of Tau, are thought to contribute to protein misfolding and subsequent formation of intracellular neurofibrillary tangles (NFTs), which are a major hallmark lesion of AD and other neurodegenerative diseases, especially frontotemporal lobar degeneration diseases [38]. Abnormal phosphorylation at specific amino acids, such as Ser^199^, Ser^202^/Ser^205^, Thr^231^, and Ser^262^, has been associated with the formation of pre-tangles in the neurons, while additional phosphorylation sites, such as Ser^422^ and Ser^396^ seem to appear later in disease progression [39,40].

#### 2.2.3. α-Syn

α-Syn is a presynaptic protein reported to be involved in endosomal formation and vesicle release at the synapse [14,41,42]. At least three isoforms of synuclein are produced through alternative splicing. The main and most investigated form is the full-length protein of 140 amino acids. α-Syn can be divided into three distinct regions: an amphipathic N-terminal region (residues 1–60), a central hydrophobic region (residues 61–95) known as non-Aβ component (NAC), which plays a central role in the aggregation of α-syn, and a C-terminal region (residues 96–140), which is acidic, unstructured and proline-rich [17]. Although the overall secondary structure of α-syn has not yet been fully determined, the N-terminal and NAC regions are believed to fold together into α-helices [17]. The acidic charge on the C-terminus of α-syn may provide higher hydrophilicity, in comparison with the N-terminus, thus allowing C-terminus to be better exposed and consequently be a better target for immunotherapy [43]. As reported, pathologic α-syn exists in insoluble forms that can aggregate into oligomers and fibrillar structures. Accumulation of pathologic α-syn in the form of Lewy bodies and Lewy neurites is a characteristic of several neurodegenerative diseases, especially PD [44,45,46]. As already mentioned, α-syn has been lately implicated in AD pathophysiology, as well [17].

### 2.3. Other Neurotherapeutic Strategies Targeting Aβ, Tau, and α-Syn

#### 2.3.1. Passive Immunotherapies

Along with the attempts to develop active immunotherapies targeting Aβ, many passive anti-Aβ immunotherapies have been investigated [5,47]. As recently reported [5], four monoclonal anti-Aβ antibodies have entered a phase III clinical trial: aducanumab, BAN2401, gantenerumab, and solanezumab. Aducanumab, a human IgG1 antibody selected through screening of memory B cells of healthy aged people which selectively targets soluble oligomers and insoluble fibrils of Aβ, has been recently approved as an immunotherapy for AD by the FDA, though there has been some controversy concerning its efficacy [20,21,22,23,24]. Candidate passive immunotherapies targeting tau [14,47] and α-syn [46] have been also reported in the literature.

#### 2.3.2. Peptides with Putative Action on Neurotoxic Aggregate-Species

Additional approaches against neurodegenerative diseases have been recently reported, which often use specific peptides to target neurotoxic aggregates, e.g., of Aβ or α-syn [48,49]; though these approaches are out of the scope of the present paper, we briefly refer here to humanin (HN), as an indicative example of neurotherapeutic peptides that may prevent Aβ aggregation. HN is a 24-mer peptide [50], probably of mitochondrial origin. As previously shown by our team with Circular Dichroism and Nuclear Magnetic Resonance studies [51], HN is unstructured and flexible in aqueous solutions, while it adopts a helical structure (Gly^5^−Leu^18^) in a less polar environment; structural findings suggest that unstructured HN may interact with different receptors, while in its helical conformation it may cross membranes and subsequently participate in more specific interactions. HN was previously identified to interact with Aβ, being capable of transforming fibrillar Aβ(1-40) to an amorphous peptide [52]. Thus, the interaction of HN with Aβ might explain protective functions of HN against neurotoxicity of Aβ [53].

## 3. Aβ-, Tau- and α-Syn Peptide Epitopes Used in Peptide-Based Vaccines for Neurodegenerative Diseases

### 3.1. Peptides Epitopes: General Concepts 

Peptide vaccines are based on specific B- (and T-) cell peptide epitopes. The epitope choice is a crucial step in the vaccine design, since the peptide epitopes should be able to induce strong, long-lasting humoral and/or cellular immunity against the biomolecular target [54]. 

A B-cell epitope is a specific fragment of the antigen/immunogen which is recognized by the B-cell receptors present on the surface of B-cells of a unique clone; B-cell epitopes are subsequently bound to the antibodies generated upon B-cell stimulation/maturation. B-cell epitopes can be linear and may be as short as pentapeptides, but they are mostly conformational [25,55]. T-cell epitopes are linear peptide fragments that can be bound to major histocompatibility complex proteins (MHC I, MHC II), through which they are presented on the surface of appropriate cells and subsequently recognized by T-cell receptors on CD8+ (cytotoxic T-cells) and CD4+ (helper T-cells), respectively [25,55]. Cytotoxic T-cell epitopes are usually 8- to 12-mer peptides, whereas helper T-cell epitopes (Th) are usually 12- to 17-mers [5,55]. 

Identifying epitopes in disease-associated immunogens is of great interest for designing epitope-based vaccines. NMR and X-ray crystallographic methods have been used for this purpose. T-cell and B-cell epitope computational prediction methods and tools (immunoinformatics), which are faster and less expensive than NMR and X-ray crystallography, have also been employed [55,56,57]. Moreover, antibodies have been often utilized as a template for vaccine design, following the concept that if a particular epitope is related to certain B- cell responses, then it will probably induce similar responses when administered in the form of a vaccine [4]. 

As already mentioned, the most significant efforts to develop peptide-based vaccines for AD and other neurodegenerative diseases have focussed on targeting specific epitopes of Aβ, tau, and α-syn (Section 3.2, Section 3.3, and Section 3.4, respectively). Special examples of how peptide-based vaccines may be advantageous over the ones based on the intact polypeptide/proteins are extensively presented (e.g., in second-generation Aβ vaccines).

### 3.2. Peptide Epitopes Used in Aβ-Vaccines

The first therapeutic approach for AD through active immunization was based on full-length pre-aggregated Aβ(1-42) [58]. However, the first clinical trials with the so-called AN1792 vaccine (Table 1), based on Aβ(1-42), failed, since meningoencephalitis appeared in some of the immunized AD patients; this severe side-effect was attributed to a cell-mediated inflammatory response caused by both, T cell epitopes of Aβ(1-42) and the adjuvant used (QS-21, Section 4.3.3). Moreover, the AN1792 vaccine induced rather insufficient antibody titer, probably due to the weak immunogenicity of Aβ [3,5,59].

Despite the serious safety issues and the rather low efficacy, results of the initial clinical trials with AN1792 along with follow-up data inspired further research toward the development of vaccines targeting Aβ. Special efforts were focused on how to avoid autoreactive T cells against Aβ and to generate relatively high titers of antibodies. Biochemical assays have identified the first N-terminal 15 amino acids of Aβ as the site of the principal B-cell epitope [60], whereas the T-cell epitopes are believed to localize in the C-terminus. Thus, second-generation Aβ vaccines were developed by selecting peptide fragments of the N terminus of Aβ, and suitably formulating [61] these fragments with foreign carriers/delivery systems and adjuvants, so as to enhance the immunogenicity of B-cell epitopes [4,5]. 

Second-generation Aβ vaccines that have subsequently been tested in clinical trials (Table 1) include CAD106, ACC-001 (vanutide cridificar), Lu AF20513, UB-311, ACI-24, V-950, ABvac40, AD01, AD02, and AD03 [3,4,5,14,61,62]. CAD106 [1,3,4,5,14,61,62,63,64] is based on the first 6 amino acids of Aβ, ACC-001 [1,3,5,14,61,62,65] is based on the first 7 amino acids, Lu AF20513 [3,4,5,61,62] is based on the first 12 amino acids, UB-311 [3,4,5,14,61,62,66] is based on the first 14 amino acids, and ACI-24 [1,3,5,14,61,62] is based on the first 15 amino acids, whereas V-950 also employs an N-terminal Aβ peptide fragment [3,61,62], reported to be Aβ(1-15) [66]. On the other hand, it should be noticed that ABvac40 vaccine [1,3,4,5,14,61,67] has employed a short C-terminal fragment of Aβ, i.e., Aβ(33-40), based on the observation that antibodies raised against the C-terminus of Aβ seem to affect Aβ aggregation [67,68]. Moreover, AD01 [3], AD02 and AD03 [3,5,14,61,62,69] are based on epitopes mimicking the Aβ N-terminus [3,62]. 

Though none of the second-generation vaccines have induced meningoencephalitis, a few antibody-mediated adverse effects, such as vasogenic edema and microhemorrhages, were reported. Moreover, these vaccines have not led to any substantial clinical benefit [70] and, as reported, clinical trials are currently ongoing only for a few of them, i.e., CAD106, ACI-24, UB-311, and ABvac40 [5]. 

In addition to vaccines that have undergone clinical trials, many other peptide-based Aβ-vaccines have been developed and tested in rodents, mostly transgenic mice. Most of these vaccines focus on the Aβ N-terminus. More specifically (Table 1), Aβ(3-10) [70,71,72] and Aβ(1-6) were among the peptide epitopes used [73]; different copy numbers of the latter were used in another attempt to develop new Aβ- vaccines [74]. In addition, Aβ(1-6) and Aβ(1-15) were used for the synthesis of 4-branched multiple-antigen peptides (MAP)_4_ and subsequently administered to mice to develop Aβ specific antibodies [75]. Aβ(1-15) was also used in a cholera toxin B subunit/silkworm pupa vaccine [76] as well as in a yeast-based vaccine (Y-5A15) [77]. Aβ(1-11) was used in a combination vaccine (AV-1959R/AV-1980R) targeting both Aβ and tau [78]. Moreover, Aβ(1-11) was used in a DNA vaccine (AB-1959D), which encoded for a fusion protein containing three copies of Aβ(1-11) [79]. Aβ(1-11) was also part of a fusion protein containing the bacterial protein domain E2, (1-11)E2, which was further used in animal vaccination [80]. Vaccines based on small cyclic peptides, i.e., cyclo[Aβ(22-28)-YNGK’], cyclo[Aβ(23-29)-ΥNGK’], and cyclo[Aβ(22-29)-YNGK’], mimicking the specific molecular turn in the structure of oligomeric Aβ were reported [81]. In a recent paper, a series of several synthetic Aβ epitope-peptides, linear or cyclic, including Aβ(1-6), Aβ(1-6)_3_, Aβ(1-15), cyclo[Aβ(1-7)], cycloEP1 and cycloEP2 (where EP1 and EP2 are special peptide epitopes of Aβ oligomers, selected with phage display from random peptide libraries), have been appropriately formulated and subsequently tested in animal preclinical studies [40]. Interestingly, intact Aβ(1-42) has been specifically formulated and tested in animal preclinical studies, without inducing neuroinflammation [82]. Moreover, a DNA vaccine based on Aβ(1-42) trimer and targeting amyloid plaques and tau protein was also reported [83]. Since soluble Aβ oligomers and protofibrils are now considered as the most toxic forms of Aβ and a discontinuous conformational epitope formed by soluble Aβ oligomers might be the ideal target of an Aβ-vaccine [18,61,68], one more vaccine (AOE1) based on specific conformational epitope(s) of Aβ oligomers as an immunogen has been recently described [84]. 

### 3.3. Peptide Epitopes Used in Tau-Vaccines

Targeting therapeutically relevant epitopes on tau proteins in a safe manner is a very interesting and highly challenging objective [11]. Identification of the most effective tau epitopes for vaccine development is still a matter of debate since many modifications of tau (phosphorylation, truncation, oligomerization, etc.) have been demonstrated to contribute to neurodegenerative disease pathogenesis.

Two tau vaccines have been tested in clinical trials with AD patients, AADvac1 and ACI-35 (Table 1). AADvac1 [1,3,4,14,61,85,86,87,88] is based on tau(294-305). This sequence was identified through experimental immunization of transgenic mice with mis-disordered tau(151-391/4R) followed by isolation of antibodies and screening for in vitro disruption of tau-tau interaction [4]; tau(294-305) epitope was determined by X-ray crystallography [89] and then tested in an AD-animal model [90]. AADvac1 induces specific antibodies targeting three or four conformational epitopes on mis-disordered tau protein with an exposed microtubule-binding repeat domain (MTBR), which seems to be actively involved in tau aggregation [14,86,87]. Initial clinical trials have proved vaccine safety, thus encouraging further trials with larger numbers of participants [4]. On the other hand, vaccine ACI-35 [1,3,4,14,86,88] is based on the synthetic C-terminal peptide tau(393-408); more specifically, it consists of 16 copies of the aforementioned tau fragment, phosphorylated at Ser^396^ and Ser^404^ [4,11,86]. The rationale is that the antibodies induced will attack tau conformers containing the pathologic phosphorylation residues rather than the non-pathologic tau species [88]. A few years ago, a phase Ib/IIa trial was conducted to validate the safety, tolerability, and immunogenicity of an advanced version of this vaccine, i.e., ACI-35.030 [1,3,14].

In addition to the vaccines that have undergone clinical trials, other tau-vaccines have been developed and tested in animal preclinical studies (Table 1). More specifically, the N-terminal region tau(2-18), also known as phosphatase activating domain (PAD), has been appropriately formulated and used for vaccinating transgenic mice, since PAD becomes exposed in pathological tau and plays an essential role in tau polymerization. [34,78,91,92]. The N-terminal region of tau is also the target of many passive anti-tau immunotherapies [11,93]. On the other hand, the epitope tau(294-305), i.e., that used in vaccine AADvac-1, has been appropriately formulated (T294-HBcVLP) and the vaccine was tested in AD transgenic mice [94]. 

Many vaccines recently tested in animals target phosphorylated tau (phospho-tau) epitopes [95,96,97,98]. Pathologic phosphorylation is a crucial event in tau pathogenicity and it is believed that by eliminating such toxic tau species, the degenerative process may be blocked [99]. For example, phosphorylation of Ser^396^ may lead to a switch from an α-helix to a β-strand motif that misfolds tau and stabilizes a toxic conformation. Therefore, early prevention of Ser^396^-phosphorylation through immunotherapy might prevent the spreading of this pathologic tau species [93]. 

A series of peptides from tau protein, including the so-called T294, pTau(396-404), and pTau422, have been used in a recent preclinical study. Two phosphorylated epitopes of tau, pTau(396-404) and pTau422, along with T294, i.e., tau(294-305), were appropriately formulated using the so-called SpyCatcher/SpyTag technology and administered to AD transgenic mice; as shown, the vaccine based on pTau422 peptide alleviated cognition deficits and blocked neuropathology progression in animals [40].

In another recent preclinical study, three phosphorylated tau peptides (pTau peptides) bearing a combination of up to four AD-related epitopes were designed and synthesized. pTau30 has been phosphorylated at residues Ser^202^/Thr^205^/Ser^238^/Ser^262^; pTau31 has been phosphorylated at residues Ser^202^/Thr^205^/Ser^396^/Ser^404^; pTau35 has been phosphorylated at Ser^238^/Ser^262^/Ser^396^/Ser^404^. Mice immunization has shown that only pTau31-induced antibodies could recognize all carried four epitopes. Furthermore, pTau31 could neither elicit non-phosphorylated Tau31-specific antibodies nor stimulate tau-specific T-cell activation [97]. Moreover, other tau peptides containing tauopathy-related phosphorylated epitopes, i.e., tau(195-213)/p202/205, tau(207-220)/p212/214, and tau(224-238)/p231, were used for animal vaccination in preclinical studies, leading to results supporting the alleviation of both, tau and Aβ pathologies [95]. In a previous work, tau(379-408)/p396/404 was tested in preclinical studies leading to a reduction in both, tau and Aβ pathologies [1,100].

Overall, it should be noticed that, as mentioned, tau contains more than eighty potential serine, threonine, and tyrosine phosphorylation sites, and many phosphorylated epitopes of tau are also present in healthy human brains; therefore, careful consideration is needed as to which epitope will be chosen and targeted [88,98].

### 3.4. Peptide Epitopes Used in α-Syn Vaccines

In order to identify surface-exposed epitopes of in vitro and in vivo formed aggregates of α-syn, polyclonal IgY antibodies were raised against short linear peptides of the α-syn molecule and used in suitable immunochemical studies [101]. The N-terminal α-syn(1-10) and C-terminal α-syn (90-140) fragments were found to be exposed with ELISA experiments [101]. On the other hand, the C-terminal epitopes α-syn(111-140)/α-syn(121-140) were recognized by human anti-α-syn antibodies isolated from PD patients [102]. Moreover, the secondary structural features of α-syn in an aqueous environment have been studied with computer simulation [103]. All this information might be further exploited when designing novel peptide-based vaccines targeting α-syn.

The only α-syn vaccines that have been tested in clinical trials (Table 1) are based on short synthetic peptide fragments that mimic C-terminal residues of α-syn(110-130) (PD01A and PD03A, Affiris) [14,46,104,105,106,107,108]. PD01A and PD03A have shown promising efficacy and safety in phase I clinical trials with multiple system atrophy (MSA) patients. Interestingly, peptides from the C-terminus of α-syn have been reported to induce in vitro both helper and cytotoxic T cell autoimmune responses in patients with PD [109]; the still adequately good safety features of PD01A/PD03A, which target the α-syn C-terminus, may be attributed to two main reasons, (i) the C-terminal peptides used are too short for inducing a T-cell autoimmune response, and, (ii) the vaccines developed do not bear the exact native epitope, but rather a mimicking sequence [14,46,108]. In July 2021, the AC Immune Company announced that it would begin a phase II clinical trial of an optimized formulation of the PD01 vaccine, called ACI-7104 [108].

In addition to the aforementioned vaccines tested in clinical trials, other α-syn vaccines have been developed and evaluated in preclinical studies. The first relevant efforts used recombinant human α-syn for vaccinating a mouse model of PD [110]. Since then, a few peptide-based vaccines targeting α-syn have been developed and shown efficacy in animal preclinical studies [105], including those shown in Table 1 [43,111].

**Table 1:** Peptide-based Aβ-, tau-, and α-syn vaccines for neurodegenerative diseases: Peptide epitopes and main formulation components used.

Target/Vaccine	Peptide Epitope	Carrier Protein/Delivery System	Adjuvant	Type of Study	Reference
Aβ/AN1792	Aβ(1-42)	Pre-aggregated peptide	QS-21	Clinical	[3,5,59,61,62]
Aβ/CAD106	Aβ(1-6)	Qβ-VLPs ^1^	Alum or MF59	Clinical	[1,3,4,5,14,61,62,63,64]
Aβ/ACC-001	Aβ(1-7)	CRM197 ^2^	QS-21	Clinical	[1,3,5,14,61,62,65]
Aβ/Lu AF20513	Aβ(1-12)	Tetanus toxoid	-	Clinical	[3,4,5,61,62]
Aβ/UB-311	Aβ(1-14)	UBITh ^3^	Alum + CpG ^4^	Clinical	[3,4,5,14,61,62,66]
Aβ/ACI-24	Aβ(1-15)	Liposomes	MLPA ^5^	Clinical	[1,3,5,14,61,62]
Aβ/V950	Aβ(1-15)	ISCOMATRIX	Quil A	Clinical	[3,61,62,66]
Aβ/ABvac40	Aβ(33-40)	KLH ^6^	Alum	Clinical	[1,3,4,5,14,61,67]
Aβ/AFFITOPE AD01	Aβ N-terminus mimotope	KLH	Alum	Clinical	[3]
Aβ/AFFITOPE AD02	Aβ N-terminus mimotope	KLH	Alum	Clinical	[3,5,14,61,62,69]
Aβ/AFFITOPE AD03	Aβ N-terminus mimotope	KLH	Alum	Clinical	[3,62]
Tau/AADvac1	Tau(294-305)	KLH	Alum	Clinical	[1,3,4,14,61,85,86,87,88]
Tau/ACI-35	Tau(393-408) [p^7^396/p404]	Liposomes	MLPA	Clinical	[1,3,4,14,86,88]
α-Syn/AFFITOPE PD01A	α-syn C-terminus mimotope	KLH	Alum	Clinical	[14,46,104,105,106,107]
α-Syn/ AFFITOPE PD03A	α-syn C-terminus mimotope	KLH	Alum	Clinical	[14,46,104,105,106]
Aβ	Aβ(1-6)	BLPs ^8^ fused with peptidoglycan anchoring domain (PA)	-	Preclinical	[74]
Aβ	Aβ(1-6)	Norovirus P Particles	CpG ^5^	Preclinical	[73]
Aβ	Aβ(3-10)	KLH	CFA/IFA	Preclinical	[70,71,72]
Aβ	Aβ(1-11)	Bacterial protein domain E2	Alum	Preclinical	[80]
Aβ/AV-1959D DNA vaccine	Aβ(1-11)	MultiTEP ^9^	-	Preclinical	[79]
Aβ/Y-5A15	Aβ(1-15)	Yeast cells (EBY-100)		Preclinical	[77]
Aβ	Aβ(1-15)	Silkworm pupae	Cholera toxin B subunit	Preclinical	[76]
Aβ	Aβ(1-6), Aβ(1-15)	Multiple antigenic peptide system	CFA/IFA ^10^	Preclinical	[75]
Aβ	cyclo[Aβ(22-28)-Y ^11^NGK’] cyclo[Aβ(23-29)-YNGK’], cyclo[Aβ(22-29)-YNGK’]	Tetanus toxoid	Alum+MLPA	Preclinical	[81]
Aβ/AOE1	Oligomer-specific Aβ mimotope peptide	Yeast cell (EBY-100)	-	Preclinical	[84]
Aβ	Aβ(1-42)		CFA+bvPLA2 ^12^	Preclinical	[82]
Aβ/ DNA vaccine	Aβ(1-42)	Gold particles	-	Preclinical	[83]
Aβ, Tau	Aβ(1-11), Tau(2-18)	MultiTEP	Advax ^13^+CpG	Preclinical	[78]
Aβ, Tau	Linear Aβ(1-6), Aβ(1-6)_3_, Aβ(1-15), Tau(294-305), p^7^Tau(396-404), pTau422 cycloAβ(1-7), cycloEP1 ^14^, cycloEP2 ^14^	HBc-VLPs conjugated with peptides via SpyCatcher/SpyTag technology ^15^	Alum	Preclinical	[40]
Tau	Tau(2-18)	MultiTEP	Advax+CpG	Preclinical	[34,92]
Tau	Tau(294-305)	HBc-VLPs ^16^	Alum	Preclinical	[94]
Tau	Tau(175-190)[p181]	Qβ-VLPs		Preclinical	[96]
Tau	Tau(195-213) [p202/205], Tau(207-220) [p212/214], Tau(224-238) [p231]	-	CFA+pertussis toxin	Preclinical	[95]
Tau	Tau(379-408) [p396/404]		Alum	Preclinical	[100]
Tau	pTau30 [p202/205/238/262], pTau31 [p202/205/396/404] pTau35 [p238/262/396/404]	Norovirus P particles	CpG+AS01	Preclinical	[97]
α-Syn	α-Syn(85-99) α-Syn(109-126) α-Syn(126-140)	Tetanus toxoid	Quil A	Preclinical	[111]
α-Syn	middle region: C^11^GGKNEEGAPQ (PD1) N-terminal: MDVFMKGLGGC (PD2) C-terminal: CGGEGYQDYEPEA (PD3)	Qβ-VLPs	-	Preclinical	[43]

^1^ Qβ-VLP: Virus-like particles from capsid proteins of Qβ bacteriophage. ^2^ CRM197: Nontoxic mutant of diphtheria toxin. ^3^ UBITh: Two different helper T cell peptide epitopes, MvF5 Th and HBsAg3 Th. ^4^ CpG: Cytosine phosphoguanosine motif. ^5^ MLPA: Monophosphoryl lipid A. ^6^ KLH: Keyhole limpet hemocyanin. ^7^ p: Phosphorylated amino acid residue(s) at a specific site in the protein sequence. ^8^ BLPs: Bacterium-like particles. ^9^ MultiTEP: A platform composed of 12 foreign helper T (Th) cell epitopes. ^10^ CFA/IFA: Complete Freund’s Adjuvant/Incomplete Freund’s Adjuvant. ^11^ Amino acids shown with the one-letter code. ^12^ bvPLA2: Bee venom phospholipase A2. ^13^ Advax: A novel polysaccharide-based adjuvant derived from crystalline particles of delta inulin, a natural plant sugar comprised of fructose and glucose units. ^14^ EP1, EP2: Special peptide epitopes of Aβ oligomers selected with phage display from random peptide libraries. ^15^ SpyCatcher/SpyTag technology: A novel method to load particulate epitopes on virus-like particles through the formation of an iso-peptide bond between the SpyCatcher protein and SpyTag peptide. ^16^ HBc VLP: Virus-like particles from hepatitis B virus core protein.

## 4. Formulation Components of Peptide-Based Vaccines for Neurodegenerative Diseases

Although the major challenge in peptide-vaccine development is the epitope selection, the wrong formulation may render a particular vaccine insufficient. The inclusion of suitable formulation components is necessary for optimal efficacy since peptides usually elicit a weak immune response by themselves, are especially prone to enzymatic degradation, while they are unlikely to stay deposited at the administration site thus allowing interaction with the immune cells recruited on the spot. Moreover, peptide-vaccines are usually based on B-cell epitopes and the T-cell epitopes necessary for a strong immune response are usually provided by a suitable carrier protein (see Section 4.1) or through particular delivery systems (see Section 4.2), the latter being mainly defined as the technology to administer or transport vaccine components. Adjuvants (see Section 4.3), on the other hand, which are defined mainly as agents capable of enhancing the immune response, are often formulated as a part of the vaccine [54].

Focusing on peptide-based vaccines for neurodegenerative diseases, the results of the first AD vaccine entered clinical trials, AN1792, along with the nature of these diseases have paved the way for developing/selecting better formulation components. More specifically, the phase II clinical trial of AN1792 was terminated due to meningoencephalitis; this side-effect has been attributed to detrimental proinflammatory Th1 autoimmune response induced not only by T cell epitopes from Aβ(1-42), but also by QS-21 adjuvant (Section 4.3.3), a potent Th1 immune stimulator, and maybe by the detergent polysorbate 80 added to the vaccine formulation [61,112]. Another problem of the clinical trials with AN1792 was modest immunogenicity, with only a small number of subjects showing adequate anti-Aβ antibody titer, which might have affected the therapeutic effect. On the other hand, immunosenescence, i.e., the deterioration of the immune system linked to aging, is a major challenge to the development of vaccines for the elderly population and this should be considered along with poor peptide immunogenicity when designing a peptide-based vaccine against neurodegeneration [112]. Thus, the “next-generation” vaccines for degenerative diseases were based on B-cell peptide epitopes of the target molecule and used carrier proteins/delivery systems/adjuvants which would ideally switch to Th2 immune responses.

### 4.1. Carrier Proteins

Keyhole limpet hemocyanin (KLH) is a large protein of the organism *Megathura crenulata* carrying T cell epitopes, which has been widely used as a carrier protein stimulating mainly a Th2 response [14]. KLH has been used in several peptide-based vaccines for neurodegenerative diseases (Table 1). Thus, the ABvac40 vaccine is based on Aβ(33-40) conjugated to KLH [67]. AFFITOPE AD01, AD02, and AD03 are conjugated to KLH peptide vaccines [3,62,69]. The AADvac1 tau-vaccine consists of a cysteinylated 12-mer tau peptide (Tau294-305) conjugated to KLH [85,86,87,88]. PD vaccine candidates (AFFITOPE PD01A and PD03A) contain appropriate peptide epitopes conjugated to KLH [106,107]. Moreover, the Aβ(3-10)-KLH conjugate has been used for animal vaccination in recent preclinical studies [70,71,72].

The so-called cross-reacting material 197 (CRM197) carrier protein is an inactivated and non-toxic form of diphtheria toxin. CRM197 can rapidly activate CD4+ T cells by generating a multitude of Th1 and Th2 cytokines, thereby promoting the proliferation of B cells and regulating the level of antibody production [113]. In a phase II clinical trial of the AD vaccine ACC-001, multiple copies of Aβ(1-7) peptide conjugated to CRM197 were used [65].

Tetanus toxoid (TT) is used as a carrier protein possessing multiple CD4+ T-cell (Th) epitopes. In LuAF20513 vaccine, Aβ(1-12) was conjugated to two foreign Th epitopes from TT, P2, and P30, and experimental data in transgenic mice as well as in guinea pigs and cynomolgus monkeys showed that the vaccine improved the ability to elicit an effective immune response by stimulating preexisting memory Th cells, which would eventually prove to be a great advantage in vaccination of the elderly [61,114]. Moreover, in a recent preclinical study, a vaccine based on small cyclic peptides of Aβ that had been conjugated to TT could improve memory deficits in J20 mice [81]. In a previous study, a PD vaccine including three peptides of α-syn that had also been conjugated to TT was reported [111].

The UB-311 vaccine was based on two Aβ(1–14) peptides, each linked to different helper T-cell peptide epitopes (UBITh) derived from the highly immunogenic measles virus fusion protein (MvF5) and hepatitis B virus surface antigen (HBsAg3). The peptides have been mixed with polyanionic CpG oligodeoxynucleotides (Section 4.3.4), so as to form stable micrometer-sized particulates. This strategy seems to bias Th2 over Th1 type responses [66].

The multimeric protein (1–11)E2 is a fusion protein including the first 11 N-terminal residues of Aβ and the bacterial protein domain E2, which self-assembles into a 60-mer complex; (1–11)E2 has been used for vaccinating transgenic mice in a recent preclinical study [80].

The so-called multiple antigen peptides (MAPs), i.e., synthetic peptide dendrimers which contain two functional structures, a branched oligo-lysine core, and multiple copies of peptide epitopes (often, B- and T- cell ones), have been used as immunogens instead of the conventional peptide/carrier protein conjugates. In a recent study, a four-branched MAP [(MAP)_4_] was covalently coupled with Aβ linear epitopes, i.e., Aβ(1-6) or Aβ(1-15), via 6-aminohexanoic acid to enhance epitope flexibility, and the products were used for animal immunization with promising results [75].

### 4.2. Delivery Systems

#### 4.2.1. Virus-like, Bacterium-like, and Inorganic Particles

Virus-like particles (VLPs) are self-assembly systems that spontaneously form virus-shaped particles. VLPs cannot replicate due to the lack of viral genetic material, thus being biosafe. Due to their size, they can efficiently enter lymphatic vessels and reach lymph nodes. VLPs efficiently induce B-cell responses due to high-epitope density on their surface, and also T-cell responses through interactions with antigen-presenting cells (APCs). Hepatitis B virus core protein (HBc), especially the major immunodominant region (MIR) of truncated HBc, can be assembled into VLPs and has been used for the insertion of foreign epitopes. In a recent study, HBc VLPs were used as carriers of a series of Aβ and tau epitopes. More specifically, a platform for peptide vaccine preparation was first constructed by inserting the so-called SpyCatcher protein into MIR. The resulting recombinant protein, HBc-SpyCatcher, could self-assemble in VLPs and readily bind to the epitope peptides that had been conjugated to the so-called SpyTag, of peptide nature, through a glutamic acid-glutamic acid (EE)/glycine-serine-glycine (GSG) linker. The final products were used for animal immunization with promising results [40]. Previously, tau(294-305) was fused to MIR and the VLPs formed were used for animal immunization with good preliminary results, both biochemical/histochemical and functional [94]. Another VLP system is based on Qβ bacteriophage. CAD106 vaccine combines multiple copies of Aβ(1-6) coupled to a Qβ VLP [64]. Qβ VLP-based vaccines which used either tau(175-190), phosphorylated at Thr^181^ [96], or peptides from the N-terminal, middle and C-terminal region of α-syn [43] were also developed and tested in animals.

Norovirus P particles (NoV P) have been used as a carrier/delivery system of Aβ and tau peptides [73,97] in animal studies, in which high titer-specific antibodies were elicited, with no parallel autoimmune T-cell activation.

Bacterium-like particles (BLPs) can also serve as multifunctional and safe carrier/delivery systems. BLPs have the same shape and size as the living bacterium, but they consist of peptidoglycan shell particles without the intracellular content of *Lactococcus lactis* bacteria. Peptide epitopes can be specifically loaded on the surface of BLPs via fusion with a peptidoglycan anchoring domain (PA). Different copy numbers of Aβ(1-6) were fused to PA, loaded to the surface of BLPs and the BLP-based vaccines were tested in mice [74].

Gold particles were employed as a delivery system, at least according to the authors’ previous publications [115], in a DNA vaccine based on Aβ(1-42) trimer, which was tested in transgenic mice [83].

#### 4.2.2. Liposomes and Miscellaneous Other Systems

Liposomes have been widely used for peptide-vaccine delivery since they protect peptide epitopes from enzymatic degradation and can enhance antigen presentation. The ACI-24 vaccine uses Aβ(1-15) peptides anchored to the surface of a liposome as a delivery system [14,61]. ACI-35 is a liposome-based tau-vaccine candidate that contains 16 copies of a synthetic tau phospho-peptide; peptides have been suitably modified, so as to include two palmitic acid chains at each terminus and thus allow assembly into liposomes [14,88].

In the V950 vaccine, the Aβ N-terminus was chemically linked directly to ISCOMATRIX, which is a delivery system made up of cholesterol, phospholipids, and saponins as an adjuvant, probably through a lipid anchor [68].

The yeast cell is a promising vaccine carrier and adjuvant. Yeast cells can express heterologous proteins/peptides using a surface display system; the epitopes displayed on the cell surface can be efficiently recognized by antigen-presenting cells. A yeast-based Aβ vaccine (Y-5A15) composed of five copies of Aβ(1-15) that had been displayed on the surface of a yeast cell’s wall was tested in transgenic mice [77]. Another yeast-based Aβ vaccine (AOE1), in which Aβ oligomer-specific mimotope peptides had been displayed on the yeast cell surface, was tested in specific mouse models [84].

In recent animal preclinical studies, two MultiTEP epitope vaccines, AV-1959R and AV-1980R, targeting Aβ(1-11) and tau(2-18), respectively, were developed as a combined vaccination approach targeting both Aβ and tau pathologies [78]; MultiTEP-using vaccines targeting separately tau [34] or Aβ [79] have been also developed and administered to animals. The MultiTEP platform was composed of 12 foreign Th cell epitopes, including a wide array of tetanus toxin, hepatitis B, and influenza Th epitopes. In this way (i.e., by activating, besides naïve Th cells, the pre-existing memory Th cells previously generated in response to infections and/or vaccinations with tetanus toxin, hepatitis B, and influenza), a unique opportunity to overcome immunosenescence in the elderly might be eventually provided.

### 4.3. Adjuvants

Some of the characteristics that adjuvants should have to be approved for clinical use are to be non-toxic and capable of effectively stimulating humoral and cellular immune responses with no adverse reactions, induce no autoimmune or allergic responses, and elicit long-lasting immunity. In most cases, the transition from an adjuvant that is effective in animals to human trials is challenging [116]. Currently, very few adjuvants are used in FDA-approved vaccines and these include alum (see Section 4.3.1), MF59 (see Section 4.3.2), CpG ODN (see Section 4.3.4) as well as AS01, AS03, and AS04, while many others are under experimental or clinical investigation [117].

#### 4.3.1. Alum Adjuvants

Aluminum salts are the most extensively used adjuvants in vaccine formulations approved by the FDA. The types of aluminum salts, often referred to as alum, include aluminum hydroxide, aluminum phosphate, and amorphous aluminum hydroxyphosphate sulfate. Alum is known as a strong Th2 immune stimulator, and several mechanisms have been proposed to explain its stimulatory activity [117,118]. Although it is generally considered quite a good human vaccine excipient, alum has shown poor immune-stimulating activity when administered with peptide-based vaccines; a possible explanation is that alum cannot efficiently protect peptides from proteolytic degradation [119].

Despite the aforementioned controversial opinions on its efficiency, alum is currently widely used in peptide-based vaccines for neurodegenerative diseases, alone or in combination with other excipients, both at the clinical and preclinical level. Concerning clinical trials, alum has been used in the formulation of AD and PD vaccines. More specifically, in phase II clinical trials of CAD106, alum (or MF59, Section 4.3.2) was mixed with reconstituted active CAD106, and the combined product induced optimal titers of Aβ-specific antibodies, with well-acceptable tolerability [64]. UB-311 vaccine was also formulated in an alum-containing Th2-biased delivery system, in combination with CpG ODN adjuvant (Section 4.3.4) [66]. In clinical trials of AFFITOPE AD02 and AD03 vaccines, aluminum hydroxide was added as an adjuvant showing a good safety profile [62,69]. Interestingly, in a phase II trial of AFFITOPE AD02, patients showed greater benefits for the placebo formulation AD04, containing only the adjuvant [69]. Finally, in two other AD vaccines, ABvac40 and AADvac1, and two PD vaccines, AFFITOPES PD01A and PD03A, alum was added as adjuvant [67,85,106,107]. On the other hand, in a few AD vaccines preclinically tested in animals lately, alum was added as an adjuvant, either alone [40,80,94,100] or in combination with monophosphoryl lipid A [81].

#### 4.3.2. Emulsion Adjuvants

There are two types of emulsions used in vaccine formulation, oil-in-water, and water-in-oil. In these systems, immunogens are adsorbed onto the emulsion droplets, are slowly released from the depot at the injection site, and up-taken by recruited immune cells. Freund’s adjuvant leads to water-in-oil emulsions and is widely used in animal vaccine research, while it is not approved for human use. Complete Freund’s adjuvant (CFA), which is usually necessary for prime immunization and induces a Th1-dominated response, consists of heat-killed *Mycobacterium tuberculosis* in non-metabolizable oils (paraffin oil and mannide monooleate), while Incomplete Freund’s Adjuvant (IFA), which is usually employed in booster immunizations and induces a Th2-biased response, is the same with CFA but it does not contain mycobacteria. Usage of CFA in vaccine formulation has been associated with undesirable side effects, such as weight loss, leucocytosis, and granulomatous peritonitis in mice [118,120]. Nevertheless, CFA has been used in the formulation of candidate AD vaccines tested in animal preclinical studies, often in combination with IFA [70,71,72,75,82] or in combination with *Bordetella pertussis* toxin (PT), in order to induce a proinflammatory CNS milieu resembling disease background [95].

MF59, which is composed of squalene, leads to oil-in-water emulsions and is licensed in Europe as the adjuvant component of influenza vaccines. Although MF59 cannot provide a long-lived depot at the injection site, it can induce chemokine secretion and immunogen uptake by monocytes that are subsequently differentiated to dendritic cells [118]. In phase II clinical trials of CAD106, MF59 (or alum, Section 4.3.1) was used as adjuvant [64].

#### 4.3.3. Saponin Adjuvants

Saponins are natural glycoside compounds and their ability to activate the human immune system has enabled their use as vaccine adjuvants. QS-21 is a triterpene glycoside of quillaic acid isolated from the bark of the *Quillaja saponaria* Molina tree. It is typically used in absence of a carrier vehicle and is effective in aqueous solutions. QS-21 can be recognized by lectin receptors on antigen-presenting cells and also induces cytotoxic T-cell activation, Th1 cytokine production (IL-2 and IFN-γ), specific antibody responses, Th1 and Th2 immune responses, and antigen cross-presentation [121]. The first AD vaccine, AN1792, contained QS-21 as an adjuvant, which might have contributed to the development of meningoencephalitis side-effects [59,122]. QS-21 was also used as an adjuvant in ACC-001 vaccine [65]. In addition, in recent animal studies of tau-peptide-based vaccines, QS-21 was added in the form of AS02 adjuvant (QS-21 and monophosphoryl lipid A, oil-in-water emulsion), combined with CpG ODN (Section 4.3.4) [97]. On the other hand, Quil A is a highly purified saponin adjuvant also isolated from bark extract of the *Quillaja saponaria* Molina tree. It consists of a complex mixture of around 25 different saponin molecules which share a common triterpenoid backbone and induces a balanced Th1/Th2 immune response. Quil A was added as an adjuvant in the formulation of the V950 vaccine in combination with the carrier/delivery system ISCOMATRIX [61] and was also used in animal testing of α-syn-peptide vaccines [111].

#### 4.3.4. Miscellaneous Other Adjuvants

Cytosine phospho-Guanosine (CpG) motifs in bacterial DNA seem to enhance immune stimulation. The synthetic form of CpG, the CpG oligodeoxynucleotides (CpG-ODN), is composed of ODNs with a phosphorothioate backbone containing unmethylated CpG motifs. CpG ODN is an agonist of Toll-like receptor 9 (TLR9) and can induce specific humoral and cellular immune responses of mainly Th1-type, which include cytotoxic T-cell generation and IFN-γ secretion [117]. CpG ODN was used as an adjuvant in combination with alum in phase I clinical trial of UB-311 vaccine [66]. In animal preclinical studies, CpG has been used as an adjuvant either in combination with Advax [34,78].

Advax is a novel polysaccharide adjuvant based on delta inulin, which successfully enhances the immunogenicity of vaccines, including peptide-based ones, as estimated mainly through specific antibody titers, and CD4^+^/CD8^+^ T-cell immune responses, while it acts synergistically with traditional innate immune activators, such as CpG ODN. Both in preclinical studies and human clinical trials, Advax has shown a very good profile of safety, tolerability, and efficacy [123] and has been used in combination with CpG as an adjuvant in animal preclinical studies of candidate AD vaccines [34,78].

Monophosphoryl lipid A (MPLA) is a highly purified derivative of the lipopolysaccharide (LPS) component of the cell wall of *Salmonella enterica.* It stimulates T cell responses and specific antibody generation and could activate innate immunity via Toll-like receptor 4 (TLR4), thus leading to mildly inflammatory conditions which favor the generation of Th1-associated humoral responses [118,124]. MLPA was added to the formulation of ACI-24 [61] and ACI-35 [88] vaccines as well as in research preclinical studies, combined with QS-21 (as AS02 adjuvant) [97], or alum [81].

The cholera toxin subunit-B (CTB) can be used as a powerful adjuvant to generate mucosal immunity and can enhance oral immune tolerance, due to its strong affinity to the monosialoganglioside (GM1) receptor, which is primarily located on mucosal epithelial cells. Thus, it was included in the formulation components of a vaccine composed of a CTB-Aβ(1-15) fused protein expressed in silkworm pupae, which was subsequently used for oral vaccination of transgenic mice [76].

In a recent pre-clinical study, co-immunization of an AD mouse model with intact Aβ(1-42) together with bee venom phospholipase A2 (bvPLA2) showed that bvPLA2 induces regulatory T cells (CD4+ CD25+ Foxp3+ T cells, Tregs) and ameliorates AD pathology, without undesirable T cell-mediated inflammation. These findings suggest that bvPLA2 administration can improve the safety of vaccination with intact Aβ(1-42) [82].

## 5. Animal Studies as a Research Tool for the Preclinical Evaluation of the Peptide-Based Vaccines against Neurodegenerative Diseases

### 5.1. Animal Models and Immunization Schemes Used in Recent Preclinical Studies

Choosing the right animal model to test the effectiveness of a candidate human vaccine at the preclinical level is of utmost importance. Ideally, the animal model used should closely resemble the disease in humans, sharing the mode of disease onset-progression and the type of immune response elicited after vaccination. To this end, a series of animal models for AD or other neurodegenerative diseases have been created/selected and used. Focusing on studies recently reported, various transgenic mouse models including APP/PS1 [71,77]/APPswe/PS1dE9 co-expressing human APP with Swedish mutation and exon-9-deleted presenilin [72,76,95], TauP301S of AD and Frontotemporal dementia phenotype [40,94,97], 3xTg-AD involving both Aβ and p-tau pathology [70,80,82,83,100], EAE/AD deriving from APP/PS1 crossed with EAE (experimental autoimmune encephalomyelitis) [84], Tau22/5xFAD developing both pathological Aβ and tau aggregates [78], PS19 [92], J20 [81], SNCA-OVX [43], B6SJL [111], Tg2576 as well as Tg-SwDI [79], and Tg4510 [34,96] have been employed. On the other hand, a few studies have employed Balb/C or C57/BL6 mice, so as to preliminarily estimate the vaccine capability of eliciting high antibody titers [73,74,75] (Table 2).

When a new vaccine is tested in research preclinical studies, there is not a general vaccination protocol to follow; thus, the immunization scheme is mainly based on previous experience of the researchers. For this reason, there are several differences regarding the route of administration, the total number of doses administered or the time interval(s) between the “prime” dose and the “booster” one(s) among studies (Table 2). Regarding the route of administration, the majority of studies employed subcutaneous (s.c.) administration, while few others selected the intramuscular (i.m.) route [34,73,74,78,92,96]. A few studies used intradermal (i.d.) administration of DNA vaccine formulations [79,83] and one tested oral administration [76]. The total number of doses may also differ (Table 2). The majority of the reported protocols include at least three doses, with 4 to 6 doses usually selected [71,72,73,74,75,78,82,92,94,100,111]. However, as many as 14 doses [43] or an everyday oral administration protocol for a period of almost 9 months [76] have also been reported.

The interval between doses has been reported to affect vaccine efficacy, but the optimal time for boosting remains to be identified. The majority of studies employed a time interval of 2–4 weeks, but 1.5 month- [78,92] or even 5 month- [80] intervals have also been reported. The age of animals at the beginning of vaccination, which may be correlated with progress in the development of brain pathology, has been also taken into account/investigated in some studies [70,81].

### 5.2. Methods for Assessing Vaccination Efficacy

Vaccination efficacy is usually evaluated through the investigation of humoral responses, histological characteristics, and behavioral/cognitive changes induced in vaccinated animals (Table 2).

As a rule, in vitro immunochemical methods are employed for evaluating humoral immune responses. ELISA remains the “golden standard” technique employed in all studies to verify the presence, determine the avidity (titer) and investigate the characteristics of immunogen-specific antibodies in the sera of immunized animals. Concerning characteristics of the antibodies elicited, it should be noticed that although a high titer is considered a prerequisite for clinical efficacy, a solid correlation between these two parameters remains to be unequivocally proved [5,80]. ELISA has also been used, although more rarely, for isotyping the developed antibodies [40,75,92,94], since the antibody class/subclass may provide interesting information concerning Th1/Th2 immune response [5]. Antibodies were also characterized with dot blot [75,92]. On the other hand, the presence and/or levels of key biomarkers in tissue sections, tissue lysates, or biological fluids is often monitored with immunohistochemistry, Western blot, or ELISA upon completion of vaccination. Another parameter often investigated is the pattern of cytokine production after vaccination, which is usually monitored with ELISpot [34,73,74,83,97,111].

In vivo behavioral analysis tests are usually employed to give insight into the vaccine effects on the cognitive functions of the vaccinated animals [125]. The most commonly used among them include the T maze/Y maze tests, used for assessing the spatial short-term memory and alternation of behavior, the Morris Water Maze (MWM) test, a robust and reliable test of spatial learning for rodents that is strongly correlated with hippocampal synaptic plasticity, the Novel Object Recognition (NOR) test, a relatively low-stress, efficient test for learning and memory in mice, appropriate for the detection of neuropsychological changes following pharmacological, biological, or genetic manipulations and based on the spontaneous tendency of mice to exhibit more interactions with a novel object rather than a familiar one. Other tests used complementarily with the aforementioned ones are the Open Field (OF) test [81], the Spontaneous Alternation (SA) test [81], the Novel Location Recognition (NLR) test [81], the Rotarod test [43,92,97,100], the radial arm water maze (RAWM) test [34,100], the Hind-limb clasping test [97], the grip strength test [97], the Locomotor activity test [43,100], the inverted screen test [43], the digitized gait assessment test [43], and the Experimental Autoimmune Encephalomyelitis (EAE) score for examining neurological deficits and paralysis [95].

## 6. Discussion

The development of peptide-based active immunotherapies/vaccines for the treatment of neurodegenerative diseases has been the objective of many research endeavors for the last two decades [4,25]. Since the pioneering work of Schenk et al. [58], who vaccinated PDAPP transgenic mice overexpressing mutant human APP with pre-aggregated Aβ(1-42) as a putative active immunotherapeutic strategy against AD, several other vaccines have been described; these vaccines have been mainly based on the most popular scenarios concerning the pathogenesis of neurodegeneration, i.e., amyloidosis and tauopathies [126] as well as synucleinopathies [46]. Though a monoclonal antibody, i.e., a passive immunotherapeutic agent, has been the first immunotherapy to be approved by the FDA for patients with AD [20], vaccines can eventually prove to be an advantageous treatment that can be widely used against neurodegeneration [3]. In fact, in comparison with passive immunotherapies, vaccination is not only well affordable for a large number of population groups, but it moreover induces a polyclonal response, including different antibody specificities and subclasses, which may offer better and longer-lasting protection than that of passively administered monoclonal antibodies [5]. For this reason, despite several setbacks, attempts to develop suitable vaccines for neurodegenerative diseases have practically never stopped.

The first clinical trial of a vaccine for AD (AN1792 vaccine) was based on intact Aβ(1-42); clinical trials were finally abandoned, due to the development of severe adverse effects in some of the participating patients. The adverse reactions were associated, at least partly, with a T-cell-mediated autoimmune response. In order to avoid autoimmune T-cell activation, short N-terminal Aβ-peptide fragments that corresponded to B-cell peptide epitopes were later used as antigens instead of the full-length peptide [5]. Careful selection/design of peptide (neo)epitope(s), which may ideally be recognized by the host’s B- but not T-cells [127], is a parameter of utmost importance for achieving the safety and efficacy necessary for a peptide-based vaccine against AD and other neurodegenerative diseases. In addition to the selection of suitable B-cell peptide epitopes, the safety and efficacy of a peptide-based vaccine depend on various other parameters, including the carriers/delivery systems that usually provide the external T-cell epitopes and/or facilitate final access to the in vivo biological target as well as the adjuvants ensuring enhanced immune response of the desired type, i.e., Th2-biased [5,126]. Formulation systems, in general, consist of factors that may greatly affect the final outcome of peptide-based vaccination; to support this, it is noteworthy mentioning that a new phase II clinical trial was announced for an optimized formulation of the α-syn peptide-based vaccine, PD01, a few months ago [108]. Concerning the pre-clinical in vivo animal studies, it should be noticed that specific parameters, such as the animal model, the specific vaccine excipients, and the vaccination schemes used [34,81,84,126] might have highly contributed to the overall outcome [70,72]. This issue may be responsible for the non-optimal results obtained in the subsequent trials with human subjects and should be carefully considered in future endeavors.

Future Perspectives: After two decades of research in the field, the development of successful peptide-based vaccines for neurodegenerative diseases seems to be a very ambitious, but not impossible goal to achieve. Further research on the design and selection of appropriate B-cell peptide epitopes of Aβ (including safe conformational epitopes of Aβ oligomers), tau, and α-syn, which would be capable of inducing antibodies of high titer and optimal features, and prevent any undesirable T-cell autoimmune responses, may eventually result in vaccines of great efficacy and safety. Moreover, using simultaneously multiple peptide epitopes of Aβ, tau, and α-syn may enhance the overall vaccine efficacy, in comparison with monotherapy, as several literature reports have pointed out [5,14]. Peptide-based vaccines targeting biomolecules other than Aβ, tau, or α-syn [1,14,18] may also prove to be successful, while combinations of vaccines and passive immunotherapies (antibodies) might be advantageous [14]. Research achievements in other related areas are expected to stimulate and greatly assist research toward novel peptide-based vaccines for neurodegenerative diseases. Thus, results of passive immunotherapy with anti-Aβ monoclonal antibodies are expected to provide valuable information concerning the immunochemical features, such as the epitope specificity and subclass that the vaccine-induced antibodies should ideally show [5]. Moreover, numerous peptide-based vaccines, including vaccines targeting acquired immune deficiency syndrome (AIDS), malaria, and, most recently, coronavirus disease 2019 (COVID-19), are currently under development [25], while much work has been accomplished so far toward design and development of peptide-based anticancer vaccines [128]. Interestingly, short peptides targeting Toll-like receptors have been recently proposed as promising new adjuvants in vaccine research [129,130]. In addition, deeper insight into less-studied risk factors for neurodegenerative diseases [131] and a better understanding of the biological mechanisms through which nutritional, environmental, and lifestyle parameters may affect neuropathology (similar with their influence on pathology/epidemiology of, e.g., neoplastic diseases, [132]), is expected to broaden the array of putative therapeutic targets for fighting neurodegeneration. Preliminary findings that associate gut microbiome with brain diseases [133,134], e.g., PD, through several ways including the production of proteins similar to misfolded α-syn by the intestine microbes (which may further serve as a human-protein misfolding-template), are just an example of how diverse future perspectives in this area may be. On the other hand, recent research efforts have focused on the discovery of novel biomarkers, eventually including personalized markers, for assessing onset and/or progression of neurodegenerative diseases [1,8,135]; diagnostic and/or prognostic biomarkers are especially needed in the area of synucleinopathies [14]. Moreover, much research has been recently directed toward developing improved analytical methods for reliable measurement of established biomarkers, each one alone or often in combination, in easily available biological fluids, such as plasma, urine, or saliva [1,136,137,138]. Success in the discovery of novel biomarkers and development of highly reliable and easy to perform methods for biomarker analysis will definitely facilitate the evaluation of candidate peptide-based vaccines for neurodegenerative diseases and accelerate further progress.

## 7. Conclusions

Neurodegenerative diseases are very complex and heterogeneous in nature, and their treatment remains a great challenge for the community. Interest in immunotherapeutic approaches has been rekindled after recent approval (even amid some controversy) of a monoclonal antibody as the first, passive, immunotherapy against AD by the FDA. Efforts to develop active immunotherapies, i.e., vaccines, on the other hand, have actually never been abandoned, despite initial setbacks. The vast majority of vaccines under development are peptide-based ones and employ specific peptide epitopes (or, specially designed mimotopes) of biomolecular targets associated with the neurodegeneration onset/progress, such as the polypeptide Aβ, or the proteins tau and α-syn. The overall efficacy and safety of peptide-based vaccines are greatly influenced by specific factors, such as the exact peptide epitope and the formulation components used. Further advances, including the discovery of novel biomolecular targets linked with neurodegeneration, identification of peptide-epitopes on the former through high-resolution structural biology methods or immunoinformatics, and the development of improved formulation systems are expected to accelerate/facilitate research in this particulate area of immunotherapy, aiming ultimately at clinical exploitation.

## Figures and Tables

**Table 2:** Peptide-based Aβ-, tau-, and α-syn vaccines for neurodegenerative diseases: Animal models, vaccination schemes and methods for evaluating efficacy reported in recent preclinical studies.

Target	Peptide Epitope	Animal Model	Vaccination Scheme (Administration Route/Doses/Time Intervals)	Methods for Evaluating Vaccination Efficacy	Reference
Aβ	Aβ(1-6)	C57/BL6 mice	i.m. ^1^/x4/2 w intervals	In vitro: ELISA; ELISpot; MTT ^2^ assay	[74]
Aβ	Aβ(1-6)	C57/BL6 mice	i.m./x4/2 w intervals	In vitro: ELISA; ELISpot	[73]
Aβ	Aβ(3-10)	APPSwe/PSEN1dE9- mice	s.c. ^1^/x6/ 2 w intervals	In vitro: ELISA; immunohistochemistry; Western Blot; TUNEL ^3^ staining; ROS ^4^ staining	[72]
Aβ	Aβ(3-10)	3xTg-AD mice	s.c./x7/2 w or 4 w intervals	In vitro: ELISA; immunohistochemistry; Western Blot In vivo: MWM test	[70]
Aβ	Aβ(3-10)	APP/PS1 mice	s.c./x5/ 2 w or 4 w inter-vals	In vitro: ELISA; immunohistochemistry In vivo: MWM test	[71]
Aβ	Aβ(1-11)	3xTg-AD mice	s.c./x3/4 m or 5 m intervals	In vitro: ELISA; immunohistochemistry; Thioflavin S staining	[80]
Aβ	Aβ(1-11) DNA vaccine	Tg2576 mice Tg-SwDI mice	i.d. ^1^/x4/ 2 w or 4 w intervals	In vitro: ELISA; immunohistochemistry In vivo: MRI^5^ imaging	[79]
Aβ	Aβ(1-15)	APP/PS1 mice	s.c./x3/ 2 w intervals	In vitro: ELISA; immunohistochemistry; Western Blot In vivo: Spontaneous Y maze test; NOR ^6^ test; MWM ^7^ test	[77]
Aβ	Aβ(1-15)	APPSwe/PSEN1dE9- mice	Orally/every day for ~9 months	In vitro: ELISA; Immunohistochemistry In vivo: water maze test	[76]
Aβ	Aβ(1-6), Aβ(1-15)	Balb/C mice	s.c./x4/2 w intervals	In vitro: ELISA; Dot Blot; Thioflavin T staining; TEM ^8^ scanning	[75]
Aβ	cyclo[Aβ(22-28)-Y^9^NGK’], cyclo[Aβ(23-29)- YNGK’], cyclo[Aβ(22-29)- YNGK’]	J20	s.c./x3/1 m intervals	In vitro: ELISA; immunohistochemistry In vivo: OF ^10^ test; SA ^11^ test; NOR test; NLR ^12^ test; MWM test	[81]
Aβ	Oligomer-specific Aβ mimotope peptide	EAE/AD mice	s.c./x5/2 w intervals	In vitro: ELISA; Immunohistochemistry In vivo: MWM test; Y maze test; NOR test	[84]
Aβ	Aβ(1-42)	3xTg-AD mice or Neuroinflammation model in C57/BL6	s.c./x6/2 w intervals	In vitro: ELISA; immunohistochemistry; Flow cytometry In vivo: MWM test; PET ^13^ scanning	[82]
Aβ	Aβ(1-42) DNA vaccine	3xTg-AD mice	i.d./x13/ 2 w or 6 w intervals	In vitro: ELISA; ELISpot; immunohistochemistry; Western Blot	[83]
Aβ, Tau	Aβ(1–11), Tau(2–18)	Tau22/5xFAD bigenic mice (T5x)	i.m./x4/1 m or 1.5 m intervals	In vitro: ELISA; immunohistochemistry; Western Blot; SPR ^14^ biosensor	[78]
Aβ, Tau	Linear Aβ(1-6), Aβ(1-6)_3_, Aβ(1-15), Tau(294-305), p ^15^Tau(396-404), pTau422 cycloAβ(1-7), cycloEP1 ^16^, cycloEP2 ^16^	BALB/c mice and TauP301S mice	s.c. 3 times at 2 w intervals s.c. 4 times at 2 w intervals	In vitro: ELISA; immunohistochemistry In vivo: Forced Y maze test; NOR test; MWM test	[40]
Tau	Tau(2-18)	Tg4510 mice	i.m./x7/2 w or 4 w intervals	In vitro: ELISA; ELISpot; immunohistochemistry; Western blot In vivo: NOR test; RAWM ^17^ test	[34]
Tau	Tau(2-18)	PS19 mice	i.m./x4 times/1 m, 1.5 m, or 2.5 m intervals	In vitro: ELISA; immunohistochemistry; Western blot, Dot Blot, confocal microscopy In vivo: Rotarod test; Y-maze test; NOR test; NPR ^18^ test	[92]
Tau	Tau(294-305)	TauP301S mice	s.c./x4/2 w or 3 w intervals	In vitro: ELISA; immunohistochemistry; Western blot In vivo: Forced Y maze test; Spontaneous Y maze test; NOR test; MWM test	[94]
Tau	Tau(175-190)[p181]	Tg4510 mice	i.m./x3 times/2 w intervals	In vitro: ELISA; immunohistochemistry; Western blot In vivo: NOR test; MWM test	[96]
Tau	Tau(195-213) [p ^15^202/205]; Tau(207-220) [p212/214]; Tau(224-238) [p231]	APPSwe/PSEN1dE9 mice	s.c./x2/1 w interval	In vitro: ELISA; immunohistochemistry; immunoblot In vivo: T maze test; MWM test; EAE ^19^ score	[95]
Tau	Tau(379-408) [p396/404]	3xTg-AD mice	s.c./x4/2 w or 4 w intervals	In vitro: ELISA; immunohistochemistry; Western blot In vivo: Rotarod test; RAWM test; close field symmetrical maze; locomotor activity; traverse beam test	[100]
Tau	pTau30 [p202/205/238/262], pTau31 [p202/205/396/404], pTau35 [p238/262/396/404]	TauP301S mice	-/x4/-	In vitro: ELISA; ELISpot; immunohistochemistry In vivo: Rotarod test; hind-limb clasping test; grip strength test; kyphosis test	[97]
α-Syn	α-Syn(85-99) α-Syn(109-126) α-Syn(126-140)	B6SJL mice	s.c./x4/2 w interval	In vitro: ELISA; ELISpot; immunohistochemistry; Western blot	[111]
α-Syn	middle region: C^9^GGKNEEGAPQ (PD1) N-terminal: MDVFMKGLGGC (PD2) C-terminal: CGGEGYQDYEPEA (PD3)	SNCA-OVX mice	s.c./x3, x4, x5, x14/2 w or 4 w intervals	In vitro: ELISA; immuno-histochemistry; Western blot; AS-PLA ^20^ In vivo: Rotarod test; locomotor activity; digitized gait assessment; inverted screen test	[43]

^1^ i.m./s.c./i.d.: intramuscular/subcutaneous/intradermal route of administration. ^2^ MTT: 3-[4,5-dimethylthiazol-2-yl]-2,5 diphenyl tetrazolium bromide. ^3^ TUNEL: Terminal deoxynucleotidyl transferase dUTP nick end labeling. ^4^ ROS: Reactive oxygen species. ^5^ MRI: Magnetic resonance imaging. ^6^ NOR: Novel object recognition. ^7^ MWM: Morris water maze. ^8^ TEM: Transmission electron microscopy. ^9^ Amino acids shown with the one-letter code. ^10^ OF: Open field. ^11^ SA: Spontaneous alternation. ^12^ NLR: Novel location recognition. ^13^ PET: Positron emission tomography. ^14^ SPR: Surface plasmon resonance. ^15^ p: Phosphorylated amino acid residue(s) at a specific site in the protein sequence. ^16^ EP1, EP2: Special peptide epitopes of Aβ oligomers selected with phage display from random peptide libraries. ^17^ RAWM: Radial arm water maze. ^18^ NPR: Novel placement recognition. ^19^ EAE: Experimental autoimmune encephalomyelitis. ^20^ AS-PLA: Alpha-synuclein proximity ligation assay.
